# Immunomodulatory activity of *Dioscorea membranacea* Pierre rhizomes and of its main active constituent Dioscorealide B

**DOI:** 10.1186/1472-6882-14-403

**Published:** 2014-10-16

**Authors:** Sumalee Panthong, Srisopa Ruangnoo, Pakakrong Thongdeeying, Busarawan Sriwanthana, Arunporn Itharat

**Affiliations:** Student of Doctor of Philosophy, Faculty of Medicine, Thammasat University, Pathumthani, 12120 Thailand; Department of Applied Thai Traditional Medicine, Faculty of Medicine, Thammasat University, Pathumthani, 12120 Thailand; Department of Medical Sciences, National Institute of Health, Ministry of Public Health, Nonthaburi, 11000 Thailand; Center of Excellence in Applied Thai Traditional Medicine Research (CEATMR), Thammasat University, Pathumthani, 12120 Thailand

**Keywords:** Hua-Khao-Yen, *Dioscorea membranacea* Pierre, Immunomodulatory activity, Dioscorealide B

## Abstract

**Background:**

The rhizomes of *Dioscorea membranacea* Pierre, also called Hua-Khao-Yen by Thai name, are used as ingredients in many Thai traditional medicines for the alternative or complementary treatment of cancer and AIDs. Preliminary *in vitro* studies have indicated that *D. membranacea* extracts exhibited high cytotoxic activity with several cancer cell lines, but the underlining mechanisms are far from clear. The aims of this study were to investigate the effects of ethanolic and aqueous crude extracts from *D. membranacea* Pierre, and pure compound from *D. membranacea* Pierre, Dioscorealide B, on natural killer cells activity and on lymphocyte proliferation.

**Methods:**

Immunomodulatory activity was investigated using PBMCs from healthy donors. NK cells activity was performed by the chromium release assay using PBMCs as effector cells, and K562 cells line labelled with chromium as target cells. Lymphocyte proliferation was determined by ^3^H-thymidine uptake. The degree of activation was expressed as the stimulation index.

**Results:**

The crude ethanolic extracts of *D. membranacea* Pierre significantly stimulated NK cells activity against K562 cells line at lower concentrations of 10 and 100 ng/ml, but not at higher concentrations. The ethanolic extracts showed no observable effect on lymphocyte proliferation. The crude water extracts significantly increased NK cell activity at concentrations of 10 ng/ml, 100 ng/ml, 1 μg/ml, 10 μg/ml and 100 μg/ml, and also activated lymphocyte proliferation at concentration of 1 ng/ml, 10 ng/ml, 100 ng/ml, 1 μg/ml, 5 μg/ml, 10 μg/ml and 100 μg/ml. However, Dioscorealide B had no significant effect at lower concentrations (0–1 μg/ml and 0–0.1 μg/ml, respectively) on NK cell activity and lymphocyte proliferation. In fact higher concentrations (>10 μg/ml and >0.5 μg/ml) of Dioscorealide B cause a significant decrease in NK cell activity and lymphocyte proliferation.

**Conclusions:**

*D. membranacea* Pierre stimulated NK cells activity and lymphocyte proliferation, but Dioscorealide B either had no effect, and at higher concentrations decreased NK cell activity and lymphocyte proliferation. Our results suggest that both extracts of *D. membranacea* Pierre significantly increases immune function, but the underlining mechanism is not clearly understood.

## Background

The immune system is the body’s defense against microbes and foreign antigens. In recent years many plant extracts have been evaluated for their immunomodulatory activity, by assessing stimulation of PBMC proliferation [1] and increased secretion of the cytokines, interferon (IFN-γ) and tumor necrosis factor (TNF-α) [2], since both lead to the enhancement of the body’s response to disease [3]. Natural Killer (NK) cells combat viruses and tumor cells as the body’s first immune response to such stimuli. NK cells can exert direct cytotoxic activity against tumor cells (innate system), and also stimulate production of cytokine (adaptive system). Therefore there is considerable interest in discovering immunomodulatory agents that can enhance the body’s immunity to a variety of diseases.

Several studies in recent years have demonstrated the immunomodulating effects of crude extracts of traditional herbal medicines [4–6], including Traditional Thai medicine. Such studies have identified potentially useful immunostimulatory and immune-suppressive plant extracts, in some case pure lead compounds.

*D. membranacea* Pierre, is commonly known as Hua-Khao-Yen, is a Thai medicinal plant used by folk doctors as an ingredient of herbal formulations used in the treatment of cancer patients [7]. The water extract of rhizome of *D. membranacea* Pierre was found to be highly active against breast cancer cell lines, and ethanolic extracts were shown to have a greater effect against lung, colon and prostate cancer cell lines, with no observable cytotoxicity against normal cell lines [8–10]. Additional the ethanolic extract of *D. membranacea* Pierre was reported to be anti-allergenic against β-hexosaminidase release as a marker of degranulation in RBL-2H3 cells, with an IC_50_ value of 37.5 μg/ml. Dioscorealide B, a naphthofuranoxepin compound isolated from the ethanolic extract *D. membranacea* Pierre, was found to suppress production of β-hexosaminidase, TNF-α, and IL-4 in RBL-2H3 cell line [11]. Furthermore, the ethanolic extract of *D. membranacea* Pierre showed antimalarial activity against K1 and 3D7 *Plasmodium falciparum* with selectivity index of 29.4 and 24.2, respectively [12]. Dioscorealide B, was also reported to against lung cancer cell line (COR-L23 and A549) [10]. Dioscorealide B against two human breast cancer cell lines; MCF-7 and MDA-MB 468 (IC_50_ = 2.76 and 9.93 μM, respectively) [13]. Moreover, Dioscorealide B also suppressed NO production and mRNA expression of iNOS, IL-1β, IL-6, and IL-10 in RAW264.7 macrophages [14].

The purpose of this present study was to investigate the effect of *D. membranacea* Pierre crude ethanolic and aqueous extracts, and Dioscorealide B, on PBMC proliferation and NK cell activity against K562 cell lines using PBMCs from healthy donors. Such detailed studies have not previously been reported, but they are necessary to in order to obtain data that might in future help in the development of immunomodulatory agents from Thai medicinal plants.

## Methods

### Reagents

Penicillin-streptomycin solution and trypan blue were purchased from Sigma-Aldrich Co. (St. Louis, MO, USA). RPMI 1640 medium, fetal bovine serum (FBS), L-glutamine and 4-(2-hydroxyethyl)-1-piperazineethanesulfonic acid (HEPES) were purchased from Invitrogen Life Technologies Inc. (Carlsbad, CA, USA). Ficoll-Hypaque mixture (Isoprep) was purchased from Robbins Scientific Corporation (Sunnyvale, CA, USA). ^3^H-thymidine radio nucleotide and Na_2_^51^CrO_4_ radio nucleotide were purchased from PerkinElmer (Waltham, Massachusetts, USA). Dimethyl sulphoxide (DMSO) and other solvents were obtained from RCI Labscan Limited (Bangkok, Thailand).

### Cell and cultures

K562 (ATCC CCL-243), human erythroleukemic cell lines, were purchased from the American Type Culture Collection (ATCC; Manassas, VA, USA). The cells were cultured in RPMI 1640 medium with 10 mM HEPES, 2 mM L-glutamine, 10% Fetal bovine serum, 100 U/ml of penicillin, and 100 μg/ml of streptomycin, and incubated at 37°C in an atmosphere containing 5% CO_2_ for three days.

### Plant material and preparation of crude extracts

The rhizomes of *D. membranacea* Pierre were collected by a Thai folk medicine practitioner at Amphor Patue, Chumporn Province, Thailand in February 2011. Their identity was confirmed as *D. membranacea* Pierre by comparison with authentic voucher specimens (number SKP A062041305) that is kept in the herbarium of Southern Center of Thai Medicinal Plants, Faculty of Pharmaceutical Sciences, Prince of Songkla University, Songkla, Thailand. The rhizomes were cleaned, cut into small (1 cm × 1 cm) pieces, dried in oven at 50°C (48 hours), and milled into a 40 mesh particle size powder. For preparing the ethanolic extract, dried plant powder (1 kg) was macerated with 95% ethanol (1.5 L) for 3 days at room temperature. The water extract was obtained by boiling the dried powder (1 kg) in water (2 L) for 30 minutes. After filtration (Whatman No.1 filter), the solvent and aqueous phases were evaporated to dryness using a lyophilizer. The crude extracts were dried to constant weight in a vacuum desiccator over several days. The dried extracts were weighed and stored in air-tight glass containers at -20°C until required for use in the studies. The percentage yield of the ethanolic and the aqueous extracts were 4.25 and 18.08% w/w, respectively. For experiment, the ethanolic extract was dissolved with DMSO at concentration of 50 mg/ml as stock solution while the water extract was dissolved with sterile water at concentration of 50 mg/ml and then filtered through a 0.22 μM filter membrane. Both stock solution of the water and ethanolic extracts were diluted with RPMI 1640 media for cell treatment.

### Isolation of dioscorealide B from D. membranacea Pierre extract

Dioscorealide B isolation from rhizomes of *D. membranacea* Pierre and confirmation of its structure was essentially as described in a previous report [15]. Briefly, the crude ethanolic extract was subjected to silica column chromatography, using varying polarity of mobile phase mixtures and collection of fractions. Fractions containing the required compound were pooled, filtered and evaporated to dryness. Dioscorealide B was re-crystallized from methanol and identity of this compound was established by using ^1^H and ^13^C NMR in Table 1. For cell treatment, Dioscorealide B was dissolved in DMSO at concentration of 10 mg/ml as stock solution and diluted with RPMI 1640 media.

**Table 1:** **The** ^**1**^ **H and** ^**13**^ **C NMR data of Dioscorealide B**

Position	Dioscorealide B (in DMSO-d _6_)		
	^1^H(mult, J in Hz) ^a^	^13^C	HMBC (c→H)
1		168.9	
1a		114.9	H-3
1b		136.8	H-2
2	7.76 (d, 8.5)	121.2	
3	7.88 (d, 8.5)	128.2	H-4
3a		138.5	H-2
3b		116.3	H-3,H-4
4	7.36 (d, 2.5)	103.9	
5		162.2	5-OCH_3_
6	6.88 (d, 2.5)	110.7	H-4
6a		152.1	
8	6.03 (s)	94.1	
9		141.9	9-OCH_3_
9a		130.7	
5-OCH_3_	3.96 (3H, S)	56.7	
8-OCH_3_			
9-OCH_3_	4.13 (3H,s)	60.0	

^a^Unless stated otherwise, each proton signal was integrated as one proton.

### Human subjects

Six males and six females were enrolled in the study. They are ages range from 20–45 years old. They have not been infected with hepatitis B, C virus and HIV-1. Females were not getting pregnant. Informed consent was obtained from each donor after the objective of this study had been completely explained. The study was approved by the ethical review committees of Faculty of Medicine, Thammasat University under number 119/2555.

### Isolation of peripheral blood mononuclear cells

Approximately 20 ml of blood was collected from each donor into sodium heparin blood collection tubes (BD; Franklin Lakes, NJ, USA). Peripheral blood mononuclear cells (PBMCs), from 12 healthy donors, were separated from heparinized blood using Ficoll-Hypaque density gradient [16] and were suspended in complete RPMI (RPMI-1640 medium supplemented with 10 mM HEPES, 2 mM L-glutamine, 10% Fetal bovine serum, 100 U/ml of penicillin, and 100 μg/ml of streptomycin).

The study was approved by the ethical review committees of faculty of medicine, Thammasat university under number 119/2555.

### Natural Killer cell activity assay

Natural killer cell on cytotoxicity was determined by the method of Sriwanthana and Chavalittumrong [17]. In brief, PBMCs were washed twice with 10 ml complete RPMI and adjusted to 2 × 10^6^ cells/ml in complete RPMI. PBMCs suspensions (500 μl) were placed into each polystyrene tube. To this was added media (500 μl) containing different concentrations of the crude ethanolic or water extracts, or the pure Dioscorealide B. The final concentrations of extracts were: 10 ng/ml, 100 ng/ml, 1 μg/ml, 10 μg/ml and 100 μg/ml; and for Dioscorealide B: 1 ng/ml, 10 ng/ml, 100 ng/ml, 1 μg/ml and 10 μg/ml. The cells were incubated in the presence or absence of samples at 37°C in an incubator under an atmosphere containing 5% CO_2_ for 18–24 hours. After incubation, the cultures were washed with 1 ml complete RPMI and then used as effector cells. K562 cells labeled with 100 μCi of Na_2_^51^CrO_4_ were used as target cells. These labelled target cells 100 μl (2 × 10^4^ cells/ml) were incubated with effector cells, in triplicate for each concentration, for 4 hours at 37°C in an incubator under an atmosphere containing 5% CO_2_.

The entire experiment was repeated on two further occasions (n = 3) effector cells to target cell ratio used were: 90:1, 30:1, 10:1 and 3:1. After incubation, 100 μl/well of supernatants from each well were transferred into microtiter tubes and counted in a multigamma counter (Automatic Gamma Counter, PerkinElmer, Massachusetts, USA).

The percentage of cytolysis was calculated according to the formula:


Spontaneous release was measured by incubation of target cells with media alone, while maximal release was measured by incubation of target cells with 5% Triton X-100. NK cells activity on cytotoxicity was expressed as a lytic unit (LU)/10^7^ PBMCs. One LU was defined as the number of effector cells required for 20% specific lysis of 1 × 10^4^ target cells.

### Lymphocyte proliferation assay

The lymphocyte proliferation assay was performed as described by Sriwanthana and Chavalittumrong [14]. Briefly, 100 μl PBMCs (2 × 10^6^ cells/ml) were cultured in 96 well microtiter plates with the extracts or Dioscorealide B. The final concentrations of these samples were 1 ng/ml, 10 ng/ml, 100 ng/ml, 1 μg/ml, 5 μg/ml, 10 μg/ml and 100 μg/ml, and 0.1 ng/ml, 1 ng/ml, 10 ng/ml, 100 ng/ml, 500 ng/ml, 1 μg/ml and 10 μg/ml, respectively. Incubation was at 37°C with 5% CO_2_ for 54 hours. After incubation, 20 μl ^3^H-thymidine (25 μCi/ml) was added into each 96 well plates and incubated at 37°C with 5% CO_2_ for 18 hours. Lymphocyte proliferation was determined by ^3^H-thymidine uptake, which was measured by a liquid scintillation counting using Topcount Microplate Scintillation & Luminescence Counter (Packard Instrumental Co., CT, USA). The degree of activation was expressed as the stimulation index. In any experiment, assays were carried out in triplicate for each concentration, and the entire experiment repeated on two further occasion (n = 3).

The stimulating index was calculated according to the formula:


### Statistical analysis

For statistical analysis, the values are expressed as mean ± SEM. The statistical significance was calculated by Student’s paired t-test and statistical significance was defined as p < 0.05. Data in the figures of the results represented the combined results from twelve human subjects, with each concentration performed in triplicate for each subject.

## Results and discussion

**Figure 1:**
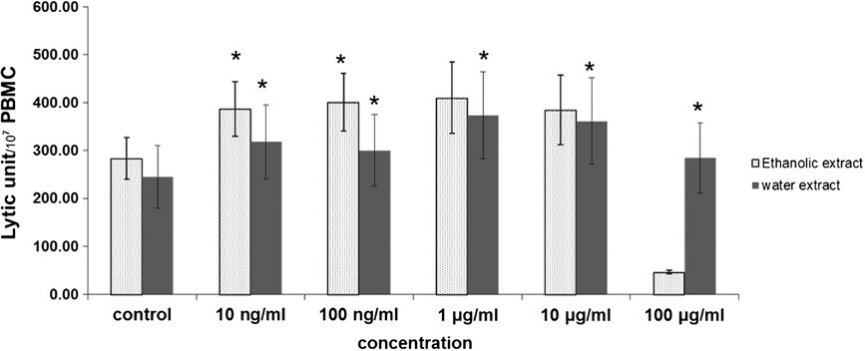
**Effect of ethanolic and aqueous extracts of the rhizomes of** ***D. membranacea*** Pierre on NK cell cytotoxicity in normal PBMC (Mean ± SEM), * p ≤0.05 vs control.

**Figure 2:**
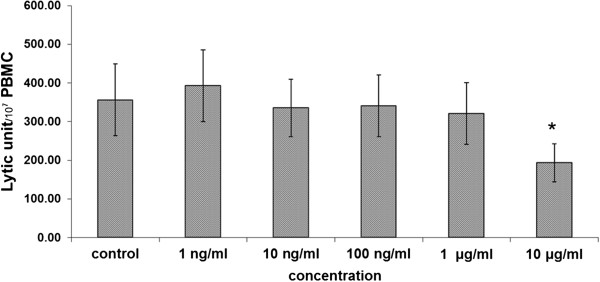
Effect of pure Dioscorealide B on NK cell cytotoxicity in normal PBMC (Mean ± SEM), * p ≤0.05 vs control.

The ethanolic extracts of *D. membranacea* Pierre significantly enhanced NK cell activity against K562 cells line at concentration of 10 ng/ml and 100 ng/ml. The higher concentrations either had no significant effect (1 μg/ml and 10 μg/ml) or decreased activity (100 μg/ml). The water extract significantly increased NK cell cytotoxicity at all concentrations (10 ng/ml to 100 μg/ml) as shown in Figure 1. Interestingly pure Dioscorealide B compound did not affect NK cell activity against K562 cells line at lower concentrations (0–1 μg/ml); in fact it significantly decreased NK cells activity at the highest concentration of 10 μg/ml as shown in Figure 2.

**Figure 3:**
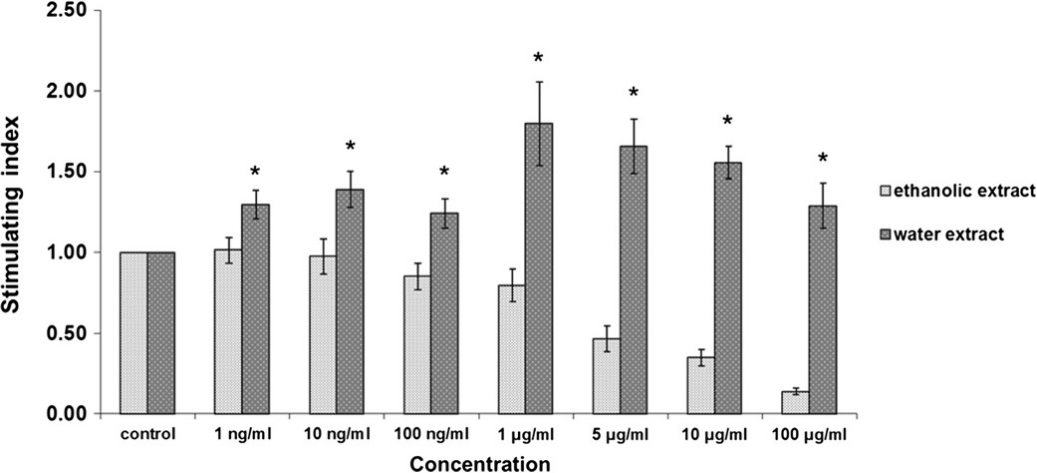
**Effects of ethanolic and aqueous extracts of the rhizomes of** ***D. membranacea*** Pierre on lymphocyte proliferation using normal PBMC (Mean ± SEM), * p ≤0.05 vs control.

**Figure 4:**
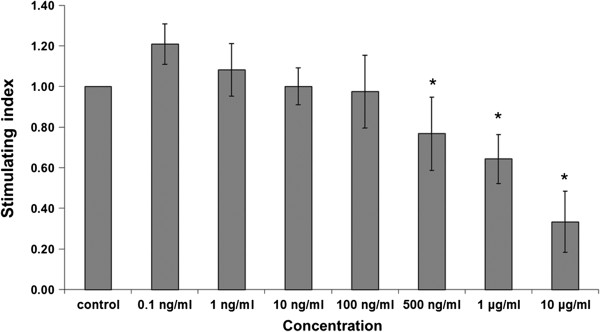
Effects of pure Dioscorealide B compound on lymphocyte proliferation using normal PBMC (Mean ± SEM), * p ≤0.05 vs control.

The results show that the ethanolic extract of *D. membranacea* Pierre had no observable significantly effect on lymphocyte proliferation, but its water extract significantly stimulated on lymphocyte proliferation at all concentrations (0–100 μg/ml) (Figure 3). On the other hand, Dioscorealide B showed no effect at lower concentrations, but was found to significantly decrease lymphocyte proliferation at higher concentrations (500 ng/ml, 1 μg/ml and 10 μg/ml) as shown in Figure 4.

There has been an interest in studying immunomodulatory activities of medicinal plants over several decades. Many medicinal plants can stimulate cellular and humoral immune systems [18]. The present study has examined the effect of *D. membranacea* Pierre and its pure active compound Dioscorealide B on NK cell cytotoxicity and lymphocyte proliferation. The roles of NK cells and lymphocytes are recognized in infected, stressed and tumour cells, and respond by directly killing these cells, and by secreting inflammatory cytokines [19].

**Figure 5:**
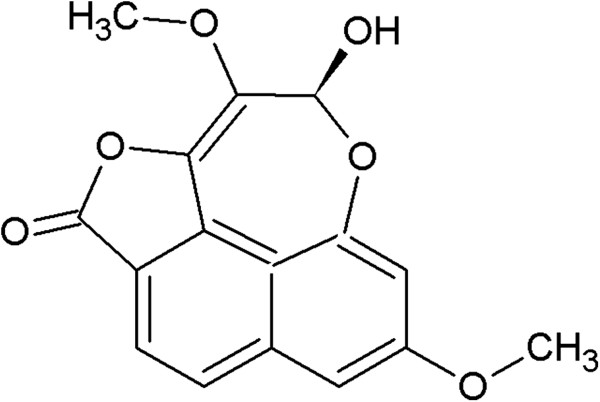
**Chemical structure of Dioscorealide B.**

**Figure 6:**
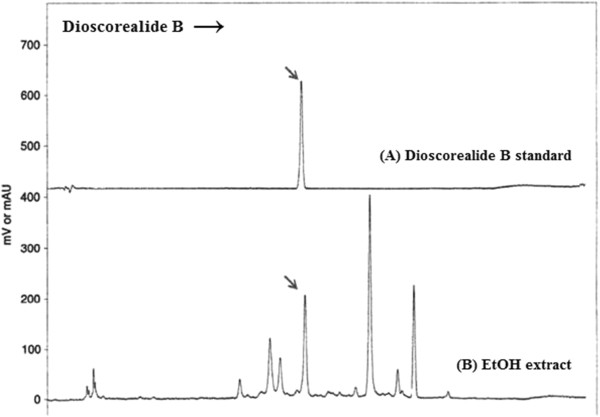
**HPLC Profile of Dioscorealide B (A) and the ethanolic extract of** ***D. membranacea*** **(B).**

The results showed that the water extract of *D. membranacea* Pierre significantly increased NK cell activity and stimulated lymphocyte proliferation, but the ethanolic extract only significantly enhanced NK cell activity. Dioscorealide B either had no effect at very low concentration or significantly decreased NK cell activity and lymphocyte proliferation at higher concentration. Dioscorealide B is a naphthofuranoxepin which found in the rhizome of *D. membranacea* as shown its structure in Figure 5. The quantitation of Dioscorealide B in the rhizome of *D. membranacea* Pierre was analysed by High Performance Liquid Chromatography-UV detection followed by the same condition in Sukkan [20]. Dioscorealide B content was calculated for standard curve which was standard curve between area under the curve (AUC) of peak on HPLC and concentration, it was determined as yield with 6.14 ± 0.13 mg/g of ethanolic extract. However Dioscorealide B was not found in the water extract. The HPLC profile of Dioscorealide B from *D. membranacea* Pierre extract as shown in Figure 6. It is possible that Dioscorealide B can exert direct toxicity on NK cells and lymphocytes, or it may induce secretion of cytokines that inhibited NK cells activity and lymphocyte proliferation. Previously, Dioscorealide B has been reported to inhibit NO production and mRNA expression of iNOS, IL-1β, IL-6 and IL-10, due to the inhibition of the upstream kinases activation, which further alleviated the NF-kB and MAPK/ERK signaling pathway in LPS-induced RAW264.7 macrophage cells [14].

To our best knowledge, this is the first time to report the effect of *D. membranacea* Pirre on NK cell activity and lymphocyte proliferation. *D. membranacea* Pirre extract may be developed as immunomodulatory agent that can improve the ability of NK cell to killed tumor and virus-infected cells in cancer and AIDS patients. Especially the water extract of *D. membranacea* Pirre has a potent effect on NK cell activity and lymphocyte proliferation at low dose to high dose. On the other hand, the ethanolic extract of *D. membranacea* Pirre should be used in lower doses because it may toxic to cells in immune system.

## Conclusions

In conclusion, our data indicated that *D. membranacea* Pierre increased NK cell activity and lymphocyte proliferation, the water extract showing better NK cell cytotoxicity and lymphocyte proliferation than its alcoholic extract. However, Dioscorealide B decreased NK cell cytotoxicity and lymphocyte proliferation at higher concentration. The results suggest that rhizomes of *D. membranacea* Pierre may contain un-identified compounds which have potent effects on the innate or the adaptive immune systems, with at present unknown mechanisms. Detailed phytochemical and bioactivity studies are necessary to identify such compounds. Furthermore, we propose that in vivo studies should be performed to evaluate the capability of *D. membranacea* Pierre to stimulate the immunity in healthy subjects.

## Authors’ information

AI is the director of the Center of Excellence in Applied Thai Traditional Medicine Research and Head of Department of Applied Thai Traditional Medicine, Faculty of Medicine, Thammasat University. We have Ph.D program on Medical Science (Nutraceutical) and Applied Thai Traditional Medicine. These programs have to responsibility on directly alternative and complementary medicine.
